# Lyme disease and Whipple’s disease: a comprehensive review for the rheumatologist

**DOI:** 10.1186/s42358-024-00359-x

**Published:** 2024-03-04

**Authors:** Henrique Ayres Mayrink Giardini, Fabricio Souza Neves, Ivanio Alves Pereira, Rafael Alves Cordeiro

**Affiliations:** 1 Rheumatology Division, Faculdade de Medicina FMUSP, Universidade de Sao Paulo, 455– 3º andar– sala 3192 Cerqueira Cesar, CEP:01246-903 Sao Paulo, SP Brazil; 2 Rheumatology Division, Internal Medicine Department, Health Sciences Center, Universidade Federal de Santa Catarina (UFSC), Florianopolis, SC Brazil; 3 Universidade do Sul de Santa Catarina (Unisul), Florianópolis, SC Brazil

**Keywords:** Lyme disease, Lyme borreliosis, Baggio-yoshinary syndrome, Whipple’s disease

## Abstract

Despite their rarity, Lyme disease and Whipple’s disease are of significant importance in rheumatology, as both can manifest as chronic arthritis, presenting challenges in the differential diagnosis of inflammatory arthropathies. In Lyme disease, arthritis typically emerges as a late manifestation, usually occurring six months after the onset of erythema migrans. The predominant presentation involves mono- or oligoarthritis of large joints, with a chronic or remitting-recurrent course. Even with appropriate antimicrobial treatment, arthritis may persist due to inadequate immunological control triggered by the disease. In contrast, Whipple’s disease may present with a migratory and intermittent seronegative poly- or oligoarthritis of large joints, preceding classic gastrointestinal symptoms by several years. Both disorders, particularly Whipple’s disease, can be misdiagnosed as more common autoimmune rheumatic conditions such as rheumatoid arthritis and spondyloarthritis. Epidemiology is crucial in suspecting and diagnosing Lyme disease, as the condition is transmitted by ticks prevalent in specific areas of the United States, Europe, and Asia. On the contrary, the causative agent of Whipple’s disease is widespread in the environment, yet invasive disease is rare and likely dependent on host genetic factors. In addition to erythema migrans in Lyme disease and gastrointestinal manifestations in Whipple’s disease, neurological and cardiac involvement can further complicate the course of both. This article offers a comprehensive review of the epidemiological, pathophysiological, clinical, and therapeutic aspects of both diseases.

## Lyme Disease

### Introduction

Lyme disease, or Lyme borreliosis, is a zoonosis caused by spirochetes of the *Borrelia burgdorferi* sensu lato group. The disease is transmitted by the bite of ticks of the genus *Ixodes* [1, 2]. *Borrelia burgdorferi* sensu stricto, which in this text will simply be called *B. burgdorferi*, is the genospecies responsible for causing the disease in North America, and the *I. scapularis* tick is its main vector. Two other genospecies recognized to cause the disease in Europe and Asia are *B. afzelii* and *B. garinii* [1, 2].

The incidence of Lyme borreliosis in the United States varies from 10 to 100 per 100,000 inhabitants, depending on the state [1]. The disease is more common in individuals aged between 5 and 15 years and after 50 years of age, and occurs more in hot months. Fortunately, mortality from the disease is low, despite the potential for morbidity [1].

During their life cycle, ticks of the genus Ixodes evolve from larvae to nymphs, and later to adult ticks. In each of these three life stages, the tick feeds only once, and can become infected from a natural reservoir in one cycle and transmit the disease in the other [1, 2]. Nymphs are the main transmitters. The natural reservoirs are wild mammals and birds, and man is an incidental host of these spirochetes [1].

Transmission to humans occurs during the bite when the tick infuses contaminated saliva into the tissue. For transmission to occur, a period of contact between tick and host is necessary, which can vary from 17 to 36 h depending on the species [2].

After the tick bite, the spirochete spreads throughout the tissue and bloodstream, stimulating the individual’s innate and acquired immune system. There are no known toxins produced by these bacteria; therefore, the host’s immune response is essential for the development of clinical manifestations [2, 3]. After contamination, *B. burgdorferi* can be isolated from the erythema migrans and the bloodstream for a few weeks [3].

### Clinical manifestations

#### Erythema migrans

Erythema migrans represents the initial manifestation in most patients: a few days after the tick bite, a localized and centrifugal erythematous rash develops, which may or may not progress to central whitening, and even without antimicrobial treatment resolves within a few weeks [2, 4, 5]. At the time the rash develops, the tick is no longer attached to the individual’s skin, and many patients may not remember the bite [4]. Low fever, arthralgia, headache, regional lymphadenopathy, and other constitutional symptoms may accompany erythema migrans [2, 4, 5]. This phase, also known as early localized infection, has fundamental importance in the diagnosis of the disease [4, 5].

#### Early disseminated infection

In this phase, which occurs days to weeks after erythema migrans, the hematogenous dissemination of spirochetes can lead to the appearance of skin lesions in other sites not affected by the tick bite, exacerbation of constitutional symptoms, and signs and symptoms of involvement of other organs, such as hepatitis, splenomegaly, carditis, and various neurological manifestations [4].

#### Carditis

A rare manifestation (< 1%), it usually presents a few weeks after erythema migrans [6, 7]. Lyme disease carditis results from the invasion of heart tissue by bacteria and the intense inflammatory process they trigger. The main and most characteristic manifestation is atrioventricular block, which can be first, second, or third degree. The severity of atrioventricular block may fluctuate. More rarely, atrial and ventricular tachyarrhythmias, endocarditis, and myopericarditis can also occur [6, 7].

If cardiac involvement is suspected (dyspnea, dizziness, syncope, palpitations, chest pain, signs, and symptoms of heart failure), ordering an electrocardiogram, Holter monitoring, and echocardiogram is recommended. Fortunately, the prognosis is usually favorable with the resolution of the block after treatment, and a permanent pacemaker is rarely necessary in these patients [6, 7].

#### Neurological manifestations

Around 15% of untreated patients may present neurological manifestations at this stage, on average four weeks after the appearance of erythema migrans [8]. The main neurological manifestations are [8, 9]:

- Cranial neuropathy: mainly of the facial nerve, which may be bilateral in a quarter of patients;
- Lymphocytic meningitis: which can present with a headache, photophobia, and neck pain; cerebrospinal fluid (CSF) analysis usually demonstrates lymphocytic pleocytosis and elevated proteins with normal glucose;
- Painful radiculopathies, sometimes with excruciating pain, often confused with radiculopathies of mechanical origin.

These neurological syndromes can occur alone or in combination and should always raise the suspicion of Lyme disease in individuals from endemic areas [8].

### Late disseminated infection

#### Arthritis

Arthritis in Lyme disease generally presents months (on average six) after the untreated acute infection, and in a few cases, it may be the only manifestation of the disease. It is more related to infection by *B. burgdorferi*, being a rare complication of other genospecies [10, 11]. It complicates around 60% of untreated individuals and typically presents as mono- or oligoarthritis, with the knee being the most affected joint. The course can be chronic or remitting– recurrent, and fever is not common [4, 10, 11].

The intraarticular dissemination of the bacteria and the consequent immunological response are responsible for the initial presentation of the disease, and most patients will respond to antimicrobial treatment. In the pre-treatment phase, it is possible to identify *B. burdorferi* genetic material in the synovial fluid, and the inflammatory process is predominantly neutrophilic [10].

Even after adequate antimicrobial treatment, some individuals do not experience remission of arthritis (< 10%) [11]. In these cases, the immune response remains uncontrolled and persistent, despite the extinction of spirochetes from the tissue. This evolution, also known as post-antibiotic Lyme arthritis, involves synovial hyperplasia and a predominance of lymphocytes, monocytes, and macrophages in the synovial fluid. The course of post-antibiotic Lyme arthritis is protracted and may persist for months to years, later resolving with or without anti-inflammatory treatment [10, 11].

#### Neurological disease

Peripheral neuropathy, predominantly sensory and axonal, usually a consequence of confluent multiple mononeuritis, can complicate the progression of Lyme disease [8, 9]. Encephalomyelitis, with cognitive symptoms, ataxia, and myelopathy can also occur [8, 9].

#### Chronic atrophic acrodermatitis

Chronic atrophic acrodermatitis is a rare late complication that can affect European patients infected with *B. afzelii*. It is characterized by the appearance of a progressive atrophic lesion and is often accompanied by peripheral neuropathy [12].

### Post-treatment Lyme disease syndrome (PTLDS)

Many patients who have had Lyme disease, even after adequate treatment and without evidence of persistent infection, develop symptoms of cognitive dysfunction, fatigue, and generalized pain [12, 13].

The Infectious Disease Society of America [14] proposed, in 2006, a case definition of PTLDS that is based on the development of the clinical symptoms described in the previous paragraph within six months of the well-established diagnosis of Lyme disease, after adequate antimicrobial treatment, with symptoms lasting more than six months.

### Diagnosis

In case of clinical suspicion, it is recommended to perform serology using two different validated methods to increase specificity (immunoenzymatic and immunoblot). It is often difficult to establish the duration of the disease based on serology alone, as both IgG and IgM can remain positive for years [15].

Carrying out serological tests before two weeks of erythema migrans may cause false-negative results. At this stage, the diagnosis must be clinical, based on the presence of a suggestive skin lesion in an individual from an endemic area [15].

Patients with neurological disease who will undergo CSF collection can perform serum and CSF serology to calculate the CSF/serum antibody index. Patients with arthritis who present positive serology and Lyme disease as one of the differential diagnoses, synovial fluid polymerase chain reaction (PCR) can be performed to assist in therapeutic decisions [15].

### Treatment

Doxycycline at a dose of 100 mg twice a day (children: 4.4 mg/kg divided into two doses per day, maximum 200 mg) represents the main oral option for treating Lyme disease. The treatment duration with doxycycline varies according to the clinical manifestation, being 10 days for erythema migrans, 14–21 days for cardiac and neurological manifestations, and 28 days for arthritis [12, 15].

In children under eight years of age, pregnant women, and nursing mothers, the decision to use doxycycline must be individualized considering the lack of robust safety studies in these populations, and alternative options can be considered [15].

For patients who require hospital admission or with cerebral or spinal parenchymal manifestations, ceftriaxone 2000 mg once a day is recommended (children 50-75 mg/kg once a day, maximum 2000 mg) for 14–21 days. Patients with the recurrence of symptoms can be treated with a second course of antibiotic therapy [15].

#### Arthritis refractory to a first course of oral antibiotics

Patients with arthritis who do not respond completely to an initial course of oral antibiotics can be retreated with oral antibiotics for 28 days or, if there was no or little response to initial treatment, intravenous ceftriaxone for 14–28 days [12, 15].

Arthritis that persists despite a course of oral antibiotic therapy and a course of intravenous antibiotic therapy is called post-antibiotic Lyme arthritis. In these patients, the therapeutic options are non-hormonal anti-inflammatory drugs, glucocorticoid infiltration, synovectomy, and immunomodulatory drugs (for example, methotrexate and hydroxychloroquine) [10–12, 15].

#### Post-treatment Lyme disease syndrome (PTLDS)

There is insufficient evidence to justify the use of prolonged and/or repeated courses of antibiotic therapy in these patients who present with nonspecific symptoms of fatigue, generalized pain, or cognitive symptoms without evidence of relapse or reinfection [12, 13, 15].

### Baggio-Yoshinari Syndrome or Brazilian Lyme Disease-like illness

Since the end of the last century, Brazilian researchers have been studying cases suggestive of Lyme disease in their country. However, the application of the Centers for Disease Control and Prevention (CDC) standard serologic tests (ELISA and Western Blot) showed low sensibility and specificity in these patients [16].

To make the matter even more complicated, to date it has not been possible to isolate* B. burgdorferi* from blood or tissue samples of national cases, and ticks of the genus *Ixodes* capable of transmitting the infection to humans have not been identified in Brazilian territory [16]. More recently, the presence of the flagellin E gene from B. *burgdorferi* sensu lato was demonstrated in biological samples from Brazilian patients by PCR [17, 18].

Faced with these difficulties, Brazilian researchers chose to name this new entity Baggio-Yoshinari Syndrome (BYS) or Brazilian Lyme Disease-like Illness. The hypotheses put forward to justify the difficulties described in the previous paragraphs were that *B. burgdorferi* adapted to Brazilian environmental conditions (climate, other species of ticks), and therefore, the previously established methods of serological determination of the infection or isolation of the bacteria would not be suitable for our reality [16]. The researchers demonstrated that considering the positivity of at least four IgG bands or two IgM bands in the WB (modified WB), combined with ELISA, it was possible to identify 65% of BYS cases [16].

It is also argued that BYS presents some different clinical characteristics from classic Lyme disease, such as a higher risk of recurrence and development of reactive phenomena. Unlike what is recommended in endemic countries, national researchers suggest that BYS cases be treated with longer courses of antibiotics (three months) [16]. However, we must keep in mind the risks related to long and repeated exposure to antimicrobials, such as the development of bacterial resistance in other species [19].

## Whipple’s Disease

### Introduction

Whipple’s disease is caused by *Tropheryma whipplei*, a slow-growing, Gram-positive, rod-shaped bacterium that belongs to the actinomycetes group [20–22]. Despite being a ubiquitous bacterium in the environment, the incidence of Whipple disease is estimated at 1 to 6 cases per ten million people per year [21]. Men of Caucasian descent are more often affected, and the average age at diagnosis is 55 years [21, 22].

The disease was described in 1907 by George Whipple. However, isolating the fastidious causative organism was a very difficult task. Only in 2000 investigators had success in cultivate the agent from a cardiac valve of a patient with endocarditis and only in 2006 it was finally cultivated from the stool of a patient with Whipple’s disease [23–25].

It is believed that contamination occurs mainly through the fecal-oral o oral-oral routes. Most individuals infected with *T*. *whipplei* will not develop the disease. Genetic factors that have not yet been fully elucidated, such as rhe presence of HLA-DRB1*13 and DQB1*06, influence the risk of developing invasive disease [21, 22]. Individuals who develop the disease have lower levels of IgG AND IgM against T. whippei asymptomatic carriers [22].

Predominantly intracellular, *T. whipplei* shows tropism for the cytoplasm of macrophages. The histopathology of duodenal samples shows mucosal infiltration by foamy macrophages that present intracellular bacteria stainable by periodic acid-Schiff (PAS) [20, 22].

### Clinical manifestations

Following initial contamination, which may be asymptomatic or manifest as gastroenteritis, pneumonia, or bacteremia, most individuals will develop a humoral and cellular immune response against T. whipplei [21].

#### Classic Whipple’s Disease

This condition is characterized by arthritis, diarrhea and signs of malabsorption [20–22]. Intermittent fever, night sweats, lymphadenopathy, and skin hyperpigmentation can also accompany these classic symptoms [26]. The progression is usually slow, with signs persisting for many years before diagnosis.

Intestinal involvement occurs in 72–81% of patients [22]. Diarrhea in Whipple’s disease typically follows a chronic course and is associated with a severe malabsorptive syndrome. This can lead to weight loss (progressing to cachexia), fatigue, abdominal pain, chronic dehydration, electrolyte disorders (hypokalemia, hypomagnesemia, hypocalcemia), anemia with iron, folic acid, and B12 deficiency, and protein-losing enteropathy with severe hypoalbuminemia and edema [26]. The cause is believed to be linked to lymphatic obstruction. Endoscopically, the mucosa may appear normal, with areas of enanthema, ulceration, or diffuse yellow-white plaques [26].

Arthritis is a common manifestation (73–80%), preceding gastrointestinal symptoms by many years [22, 27–29]. The most characteristic presentation involves migratory and intermittent seronegative oligo- or polyarthritis of large joints, often affecting the knees. This arthritis is non-erosive and accompanied by an increase in inflammatory tests. However, polyarthritis of small joints, sacroiliitis, spondylodiscitis, hypertrophic osteoarthropathy, and cases with joint erosions and deformities are also reported [22, 27–29]. Many patients with Whipple’s arthritis are initially misdiagnosed as autoimmune or autoinflammatory rheumatic conditions, with seronegative rheumatoid arthritis and spondyloarthritis being the most common [28]. Typically, these patients do not respond to glucocorticoids and immunosuppressive agents, and the disease can worsen after initiating these treatments [28, 30]. In patients with seronegative arthritis, especially when accompanied by diarrhea or other systemic symptoms, this progression can be a crucial clue to the diagnosis of Whipple’s disease.

Neurological involvement occurs in 10–43% of patients [31]. The range of neurological manifestations is extensive, including cognitive changes, dementia, psychiatric and behavioral disorders, sleep disorders (hypersomnia or insomnia), obstructive hydrocephalus, vertical gaze palsy, cerebellar ataxia, and myoclonus, among others [31–33]. Oculomasticatory myoarrhythmia, characterized by pendular nystagmus accompanied by rhythmic contractions of the masticatory muscles, occurs in approximately 20% of patients. The simultaneous presence of dementia, myoclonus, and oculomasticatory myoarrhythmia strongly suggests Whipple’s disease [31–33]. Nutritional deficiencies related to malabsorptive syndrome can also cause neurological manifestations, such as peripheral neuropathy [31].

CSF analysis may be normal or show pleocytosis and a slight increase in proteins. Brain Magnetic Resonance Imaging (MRI) is crucial in the initial evaluation and follow-up. It may be normal or show changes such as atrophy, expansive lesions, diffuse hyperintense focal lesions, and pachymeningitis, which may improve with specific treatment [21, 31, 32].

Cardiac involvement is a well-described complication of Whipple’s disease. Typically, it manifests as subacute or chronic culture-negative endocarditis, often accompanied by other cardinal symptoms like arthritis, diarrhea, and weight loss [34, 35]. Most cases involve native valves, with the aortic and mitral valves being the most affected. The prognosis is poor due to delayed diagnosis, usually confirmed by valvar tissue analysis [34].

Other reported manifestations of classic Whipple disease include pleural and pericardial effusions, lung nodules [36, 37], subcutaneous nodules [38], eye disease (orbitopathy, uveitis, keratitis) [39, 40], and thrombocytopenia [41, 42].

#### Localized chronic infection

*T. whipplei* can cause localized infection of specific organs, lacking the multisystemic clinical manifestations seen in classic Whipple’s disease. Chronic arthritis, encephalitis, and endocarditis can occur independently, without gastrointestinal disease, and with negative histopathological investigation of the duodenal mucosa [20, 22].

### Diagnosis

The most common method utilized in the evaluation of Whipple`s disease is duodenal biopsy. The typical finding is mucosal infiltration by foamy macrophages containing PAS-positive intracytoplasmic bacteria [20, 22]. However, it may be negative in patients without gastrointestinal symptoms [43, 44]. Macrophages with PAS-positive intracytoplasmic bacteria can also be identified in other tissues such as the brain, bone marrow, lymph nodes, skin, liver, and heart valves. Immunohistochemistry with specific antibodies against *T. whipplei* is more specific and sensitive than PAS staining in duodenal samples [22].

Identification by PCR has become increasingly available [43, 44]. If classic Whipple’s disease is suspected, PCR of saliva and feces can be used as a screening method, and if positive, an intestinal biopsy should be performed [20, 22]. In patients with suspected localized disease, PCR of saliva and feces may be negative, but this does not exclude the disease, and investigation of affected tissues and organs should continue. PCR can be performed on tissue samples, synovial fluid, cerebrospinal fluid, and blood [22, 43, 44].

For a definitive diagnosis of Whipple disease, it is desirable to demonstrate positivity in two of the three methodologies previously discussed: PAS-positive staining on biopsy, immunohistochemistry and/or PCR (44). In general, in patients with classical Whipple`s disease, duodenal biopsy is the most commonly used and accessible site. However, it is important to consider the possibility of a negative result, especially in localized cases.

As *T. whipplei* is an intracellular and slow-growing bacterium, culture is an expensive method and is rarely available in clinical practice. Serology is not recommended as it is often positive in asymptomatic carriers [20, 22].

### Treatment

More than one treatment regimen is described for treating Whipple disease. Among the proposed schemes, the following are two options [20, 22]:

- Ceftriaxone (2 g/day) or meropenem (1 g 3 times a day) for 14 days, followed by Sulfamethoxazole + trimethoprim for 12 months; however, Sulfamethoxazole + trimethoprim is associated with greater toxicity and resistance.
- Doxycycline 200 mg/day + Hydroxychloroquine 600 mg/day for 12–18 months; in patients with classic disease, maintain *ad eternum* treatment with doxycycline 200 g/day.

Whipple’s disease can be fatal in non-treated cases, and the risk of disease recurrence is considerable. Therefore, regular clinical monitoring and duodenal biopsy or PCR of saliva and feces should be considered in the follow-up of these patients [20, 22].

## Conclusion

Lyme disease and Whipple’s disease are infectious conditions characterized by an indolent course and multisystemic manifestations. Arthritis, presenting with diverse patterns, is a cardinal manifestation in both conditions.

Epidemiological data play a pivotal role in suspecting and diagnosing Lyme disease, given the prevalence of transmitting ticks in specific regions of the United States, Europe, and Asia. Regarding Baggio-Yoshinari Syndrome, further studies are important for the microbiological and molecular identification of the causative agent. Presently, robust scientific evidence is lacking, so we can not make specific recommendations for diagnosis and treatment.

Whipple’s disease, although rare, should always be considered in patients with chronic seronegative arthritis that does not respond to conventional immunosuppression, especially when accompanied by gastrointestinal or neurological symptoms. The exacerbation of arthritis or the emergence of other systemic manifestations after immunosuppression should also raise awareness about this diagnosis.

## Data Availability

Data sharing is not applicable to this article as no datasets were generated or analyzed during the current study.

## Abbreviations

BYS: Baggio-Yoshinari syndrome
CSF: cerebrospinal fluid
MRI: Magnetic resonance imaging
PAS: Periodic acid-Schiff
PCR: Polymerase chain reaction
PTLDS: Post-treatment Lyme disease syndrome
